# Supplementary material for: Phase-locked constructing dynamic supramolecular ionic conductive elastomers with superior toughness, autonomous self-healing and recyclability
Source: Nat Commun. 2022 Aug 18;13:4868. doi: 10.1038/s41467-022-32517-4 (PMC9388535; doi:10.1038/s41467-022-32517-4)
Supplement: Supplementary file 1 — Supplementary Information [file 41467_2022_32517_MOESM1_ESM.pdf]

Supplementary Information

Phase-locked Constructing Dynamic Supramolecular Ionic Conductive Elastomers with Superior Toughness, Autonomous Self-healing and Recyclability

Jing Chen[1], Yiyang Gao[1], Lei Shi[2], Wei Yu[1], Zongjie Sun[1], Yifan Zhou[3], Shuang Liu[4], Heng Mao[1], Dongyang Zhang[1], Tongqing Lu[3], Quan Chen[4], Demei Yu[1], Shujiang Ding[1]*

[1]School of Chemistry, Xi'an Jiaotong University, Xi'an Key Laboratory of Sustainable Energy Materials Chemistry, State Key Laboratory for Mechanical Behavior of Materials, Xi'an 710049, P. R. China

[2]School of Materials, Sun Yat-sen University, Shenzhen 518107, P. R. China

[3]State Key Lab for Strength and Vibration of Mechanical Structures, International Center for Applied Mechanics, Department of Engineering Mechanics, Xi’an Jiaotong University, Xi’an 710049, P. R. China

[4]StateKey Laboratory of Polymer Physics and Chemistry, Changchun Institute of Applied Chemistry, Chinese Academy of Sciences, Changchun 130022, P. R. China

* Corresponding author. Email: dingsj@xjtu.edu.cn

This file includes:

Supplementary Fig. 1 to 25
Supplementary Tab. 1 to 2

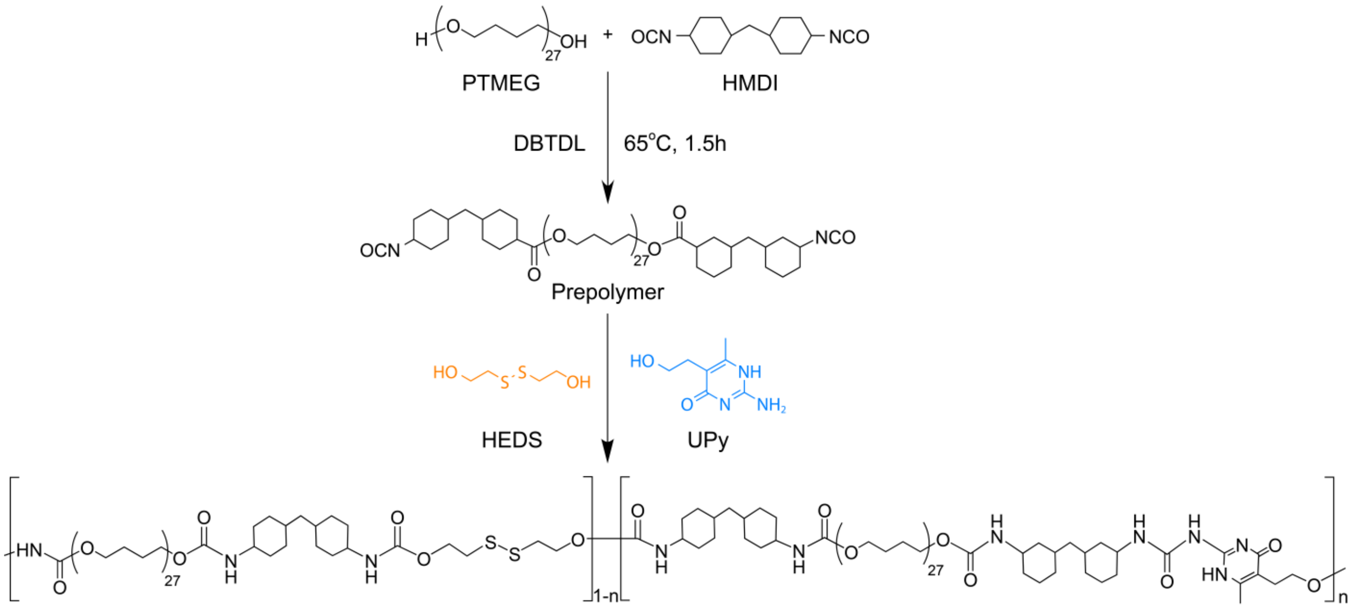

Supplementary Fig. 1. **Synthetic route of dynamic supramolecular elastomers (DSE)** via condensation polymerization.

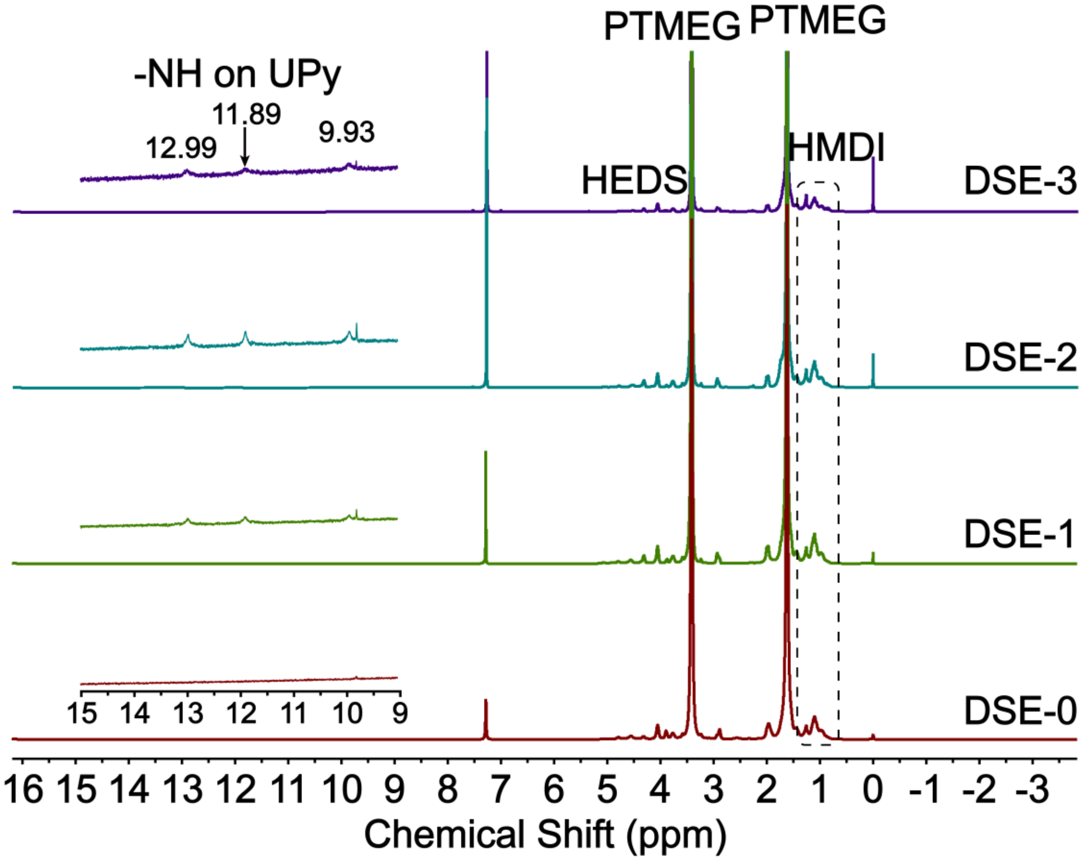

Supplementary Fig. 2. ^{1}H NMR spectra of DSE-0~3 samples in $CDCl_3$ (400 MHz). In the ^{1}H NMR spectra, the peaks of PTMEG protons appeared at 3.4 and 1.6 ppm, the peaks of HMDI unit appeared at 1.1~0.9 ppm, the peaks of HEDS unit appeared at 3.7 and 2.7 ppm, the three characteristic peaks of 12.99, 11.89 and 9.93 ppm in the downfield was observed in the DSE-1, DSE-2 and DSE-3, which demonstrated the existence of the UPy motifs.

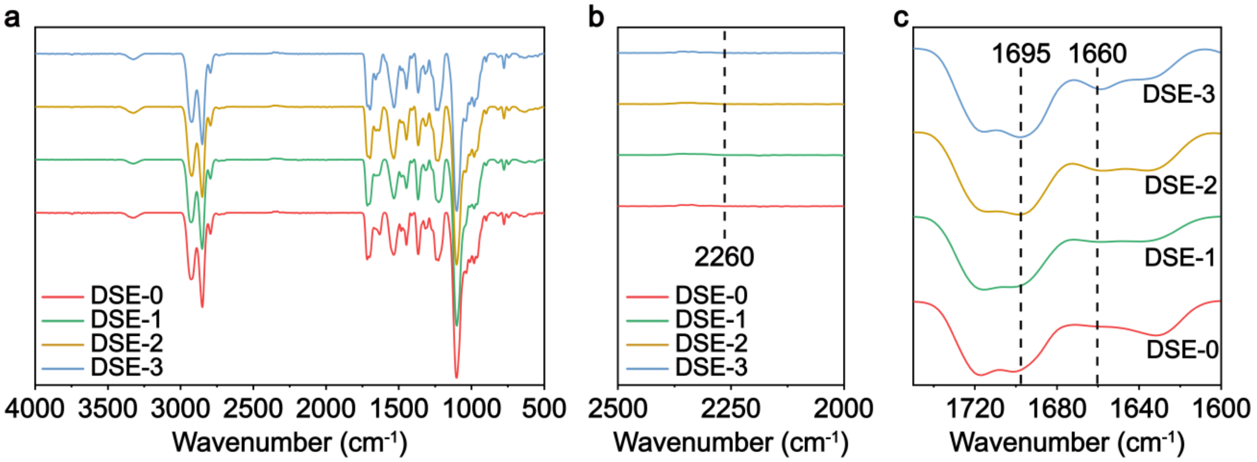

Supplementary Fig. 3. ATR-FTIR characterization of all DSE samples. (**a**) FTIR spectra from DSE-0~3 in the range of 500-4000 cm^{-1}. The existence of N-H stretching vibration around 3315 cm^{-1} indicated the formation of self-complementary N-H hydrogen bonds. (**b**) The disappearance of the peaks at 2260 cm^{-1} (N=C=O stretching band) suggested the diisocyanate monomers conduct complete reaction. (**c**) The peak associated with H-bonded C=O in urea (~1660 cm^{-1}) increases from DSE-0 to DSE-3. Similarly, the peak associated with H-bonded C=O in urethane (~1695 cm^{-1}) increases as the amount of UPy in the DSE polymer increases. The increase in the intensity of these peaks indicated the clear increase in the amount of hydrogen bonding present in the polymers as the amount of UPy increases.

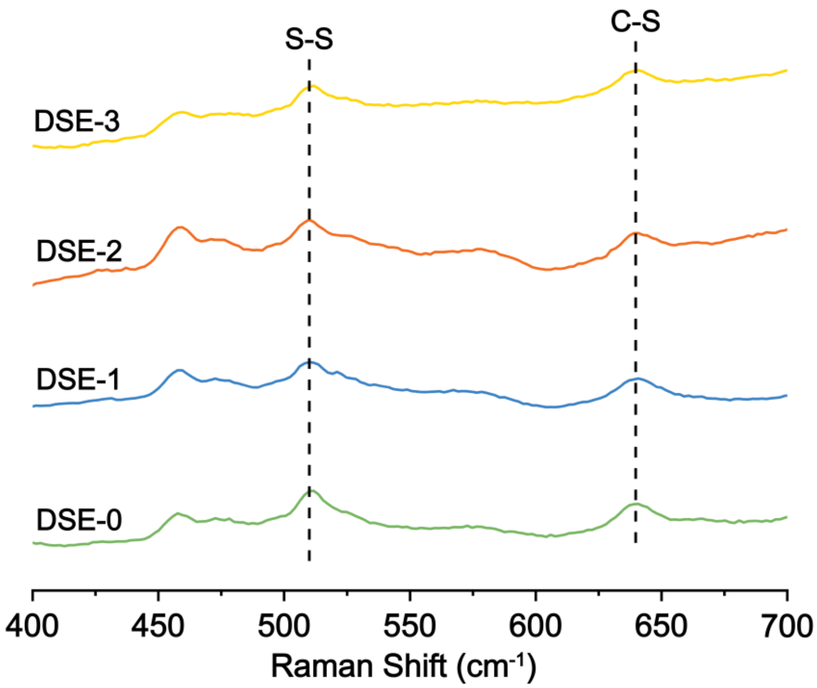

Supplementary Fig. 4. **Raman spectra investigation of all DSE samples**. The peak appears at 510 cm^{-1} is responsible for ν(S-S), the peak appears at 640 cm^{-1} is responsible for ν(C-S).

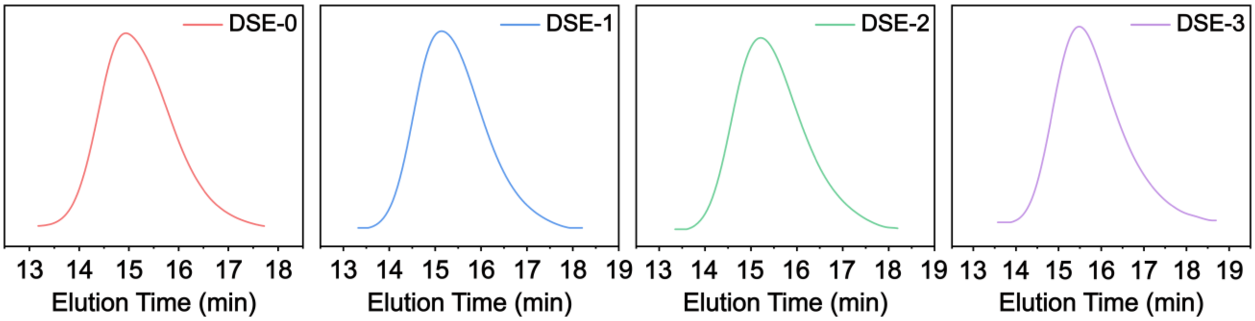

Supplementary Fig. 5. GPC elution curves of DSE-0~3 samples with THF as the eluent and PS as the standard.

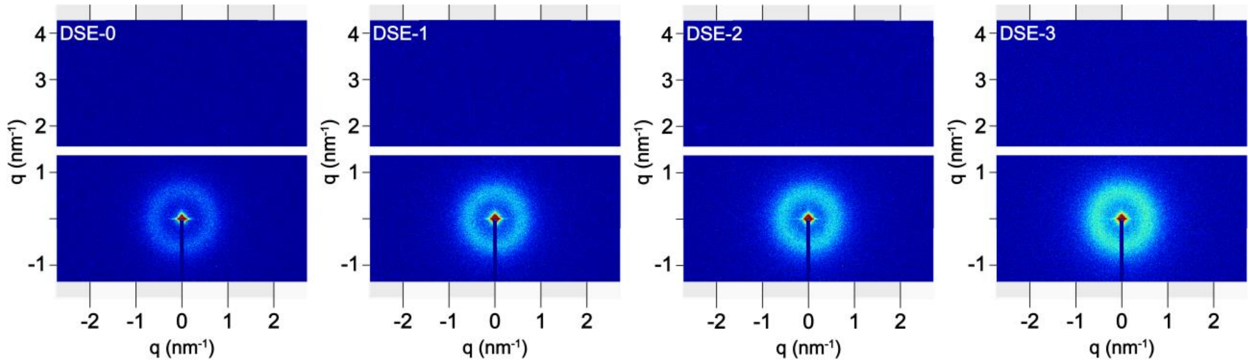

Supplementary Fig. 6. 2D SAXS images of DSE-0~3 samples. As the content of UPy motifs in the hard phase increases, the electron density contrast between the two phases increases, indicating more prominent accumulation of the hard segments.

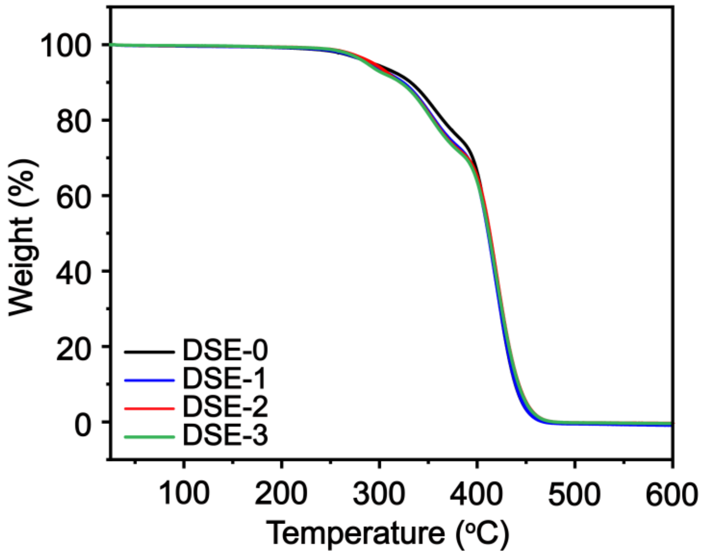

Supplementary Fig. 7. TGA traces of DSE-0~3 samples at 10 °C min^{-1} under N_2 atmosphere, which displays that the four DSE samples are thermally stable up to 275 °C.

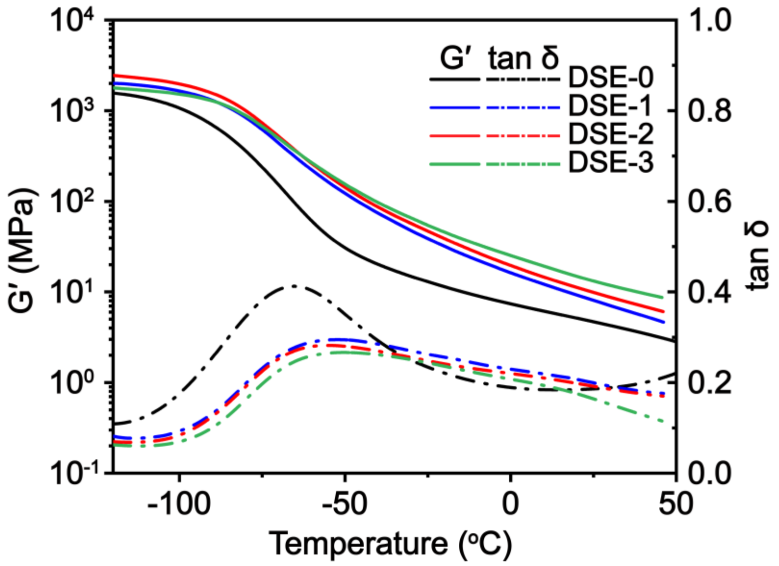

Supplementary Fig. 8. DMA curves of DSE-0~3 samples. The remarkable drops in G′ curves relating to the relaxation of soft segments were all distinct and the four strong relaxation peaks appeared in the tan δ curves, which could be assigned to Tg.

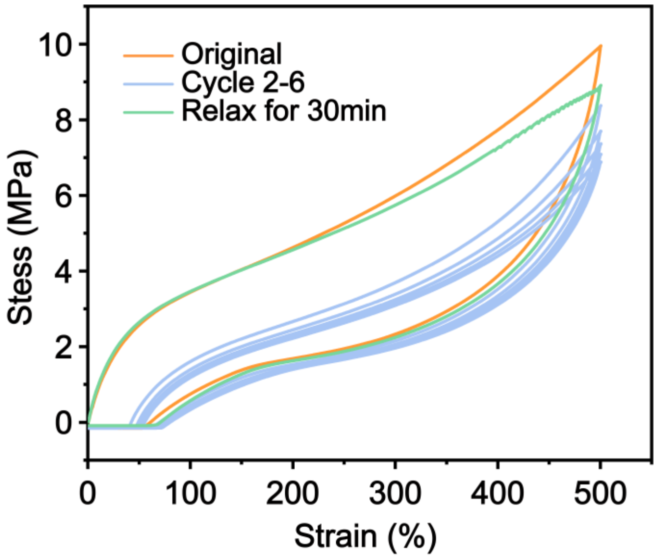

Supplementary Fig. 9. Consecutive cyclic tensile curves of DSE-3 at a strain of 500%. After relaxing for 30 min, the cycle curve was nearly overlapped with the first cycle, indicating the full self-recovery of the hysteresis area and the residual strain.

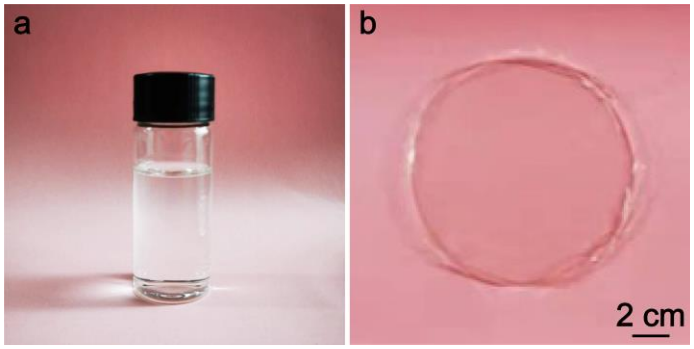

Supplementary Fig. 10. Digital photos of the prepared DSICE samples. (**a**) DSICE dissolved in anhydrous THF to colorless transparent solution. (**b**) The resulted DSICE film prepared via casting the colorless transparent solution into a glass mold and drying.

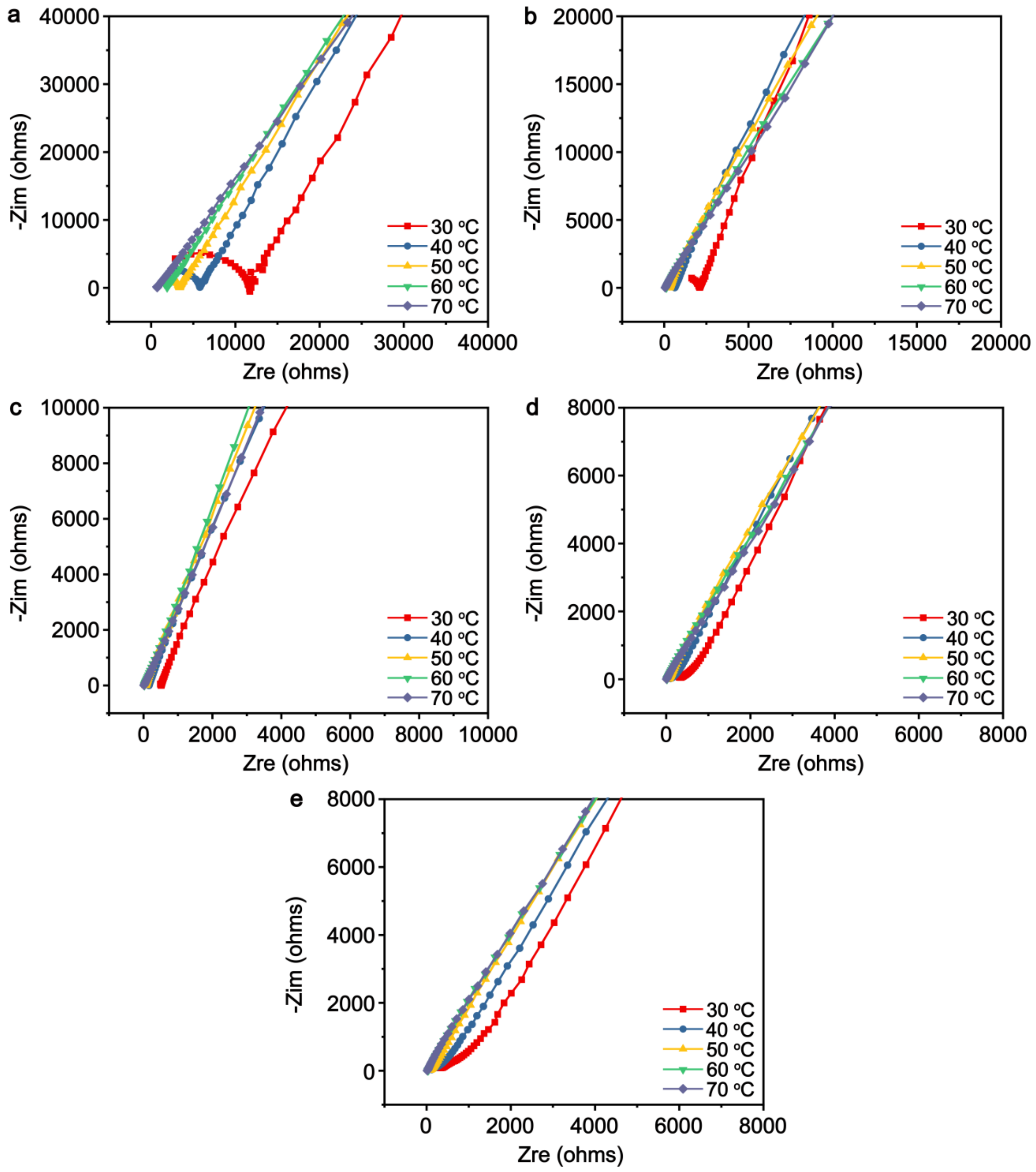

Supplementary Fig. 11. EIS spectra of DSICE-10~40 samples at different temperatures from 30 °C to 70 °C. (**a**) EIS spectra of DSICE-10. (**b**) EIS spectra of DSICE-20. (**c**) EIS spectra of DSICE-30. (**d**) EIS spectra of DSICE-35. (**e**) EIS spectra of DSICE-40.

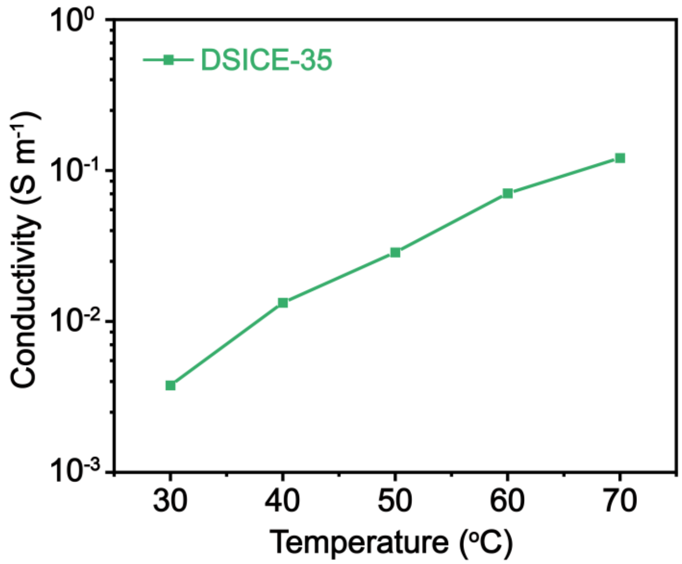

Supplementary Fig. 12. Plots of ionic conductivity versus the elevated temperatures of DSICE-35 (30 °C-70 °C). With the increasing temperature, DSICE exhibited higher conductivity, which is attributed to more intense movement of polymer chains and ions at higher temperature.

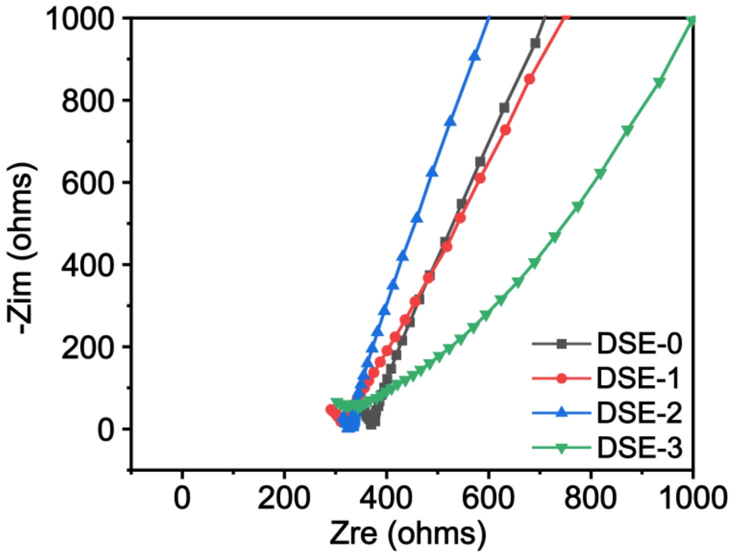

Supplementary Fig. 13. EIS spectra of DSE-0~3 samples with the same 35 wt.% LiTFSI at 30 °C. The impedance exhibited close, indicating the ionic conductivity for DSICE with the same content 35 wt.% LiTFSI in the DSE-0~3 polymers remains relatively constant (Samples size remains the same).

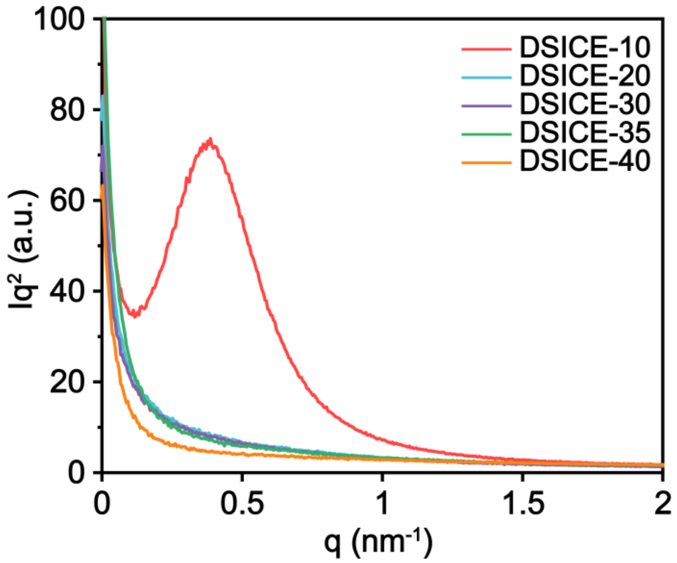

Supplementary Fig. 14. SAXS profile plots of DSICE-10~40 samples. It demonstrates that the microphase separation structure of DSICE disappeared with the increase of LiTFSI content.

The SAXS profile plots showed that the obvious microphase separation occurs when LiTFSI content is 10 wt.%, and the microphase separation disappears when the content continues to increase to more than 20 wt.%, indicating the increase of LiTFSI content hinders the accumulation of the hard phase chain domains. Hence, attenuation of hard phase chain segments stacking leads to the mechanical degradation of DSICE.

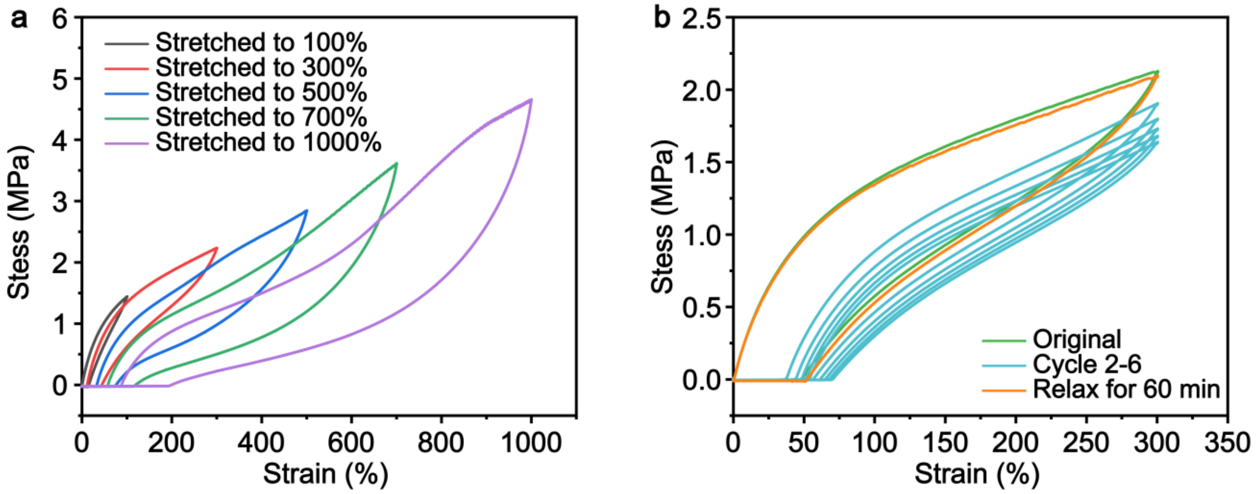

Supplementary Fig. 15. Cyclic tensile tests of DSICE-20 sample under the deformation rate of 100 mm min^{-1}. (**a**) Single cyclic stress-strain curves at different strains (100%, 300%, 500%, 700%, 1000%) in successive stretching. (**b**) Consecutive cyclic tensile curves at a strain of 300%. After relaxing for 60 min, the cycle curve was nearly overlapped with the first cycle.

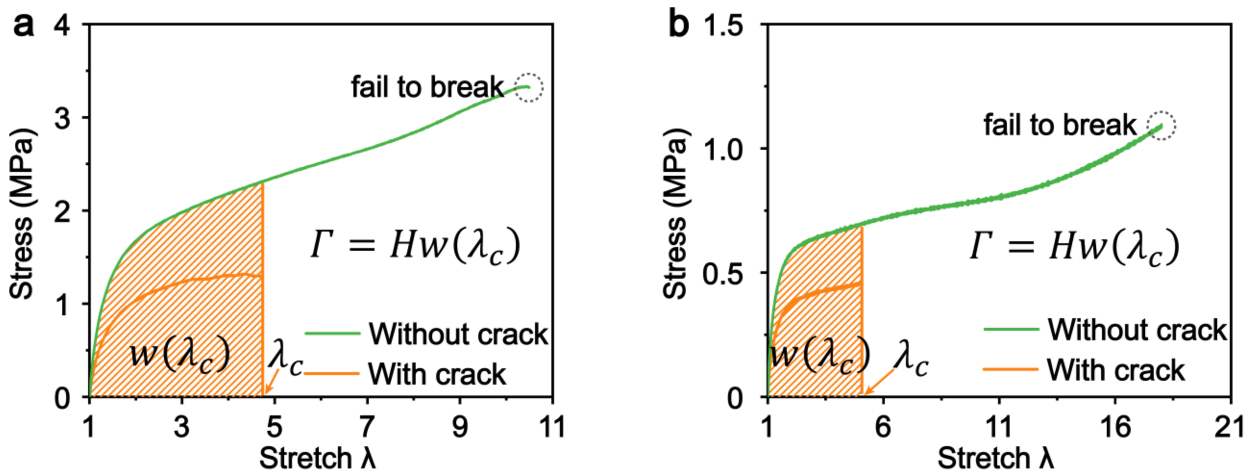

Supplementary Fig. 16. Stress-strain curves of the intact DSICE samples and one with the precut crack (gauge length: 10 mm). (**a**) The fracture energy (*Γ*) of DSICE-20 sample, calculated as 67041.68 J m^{-2}. (**b**) The fracture energy (*Γ*) of DSICE-30 sample, calculated as 23955.94 J m^{-2}.

The fracture energy value (*Γ*) is calculated by the formula:

$$\Gamma = Hw(\lambda c)$$

where λc is the critical stretch of the crack propagation, $w(\lambda c)$ is obtained by integrating the area under the stress-strain curve of the unnotched sample at the λc, H is gauge length.

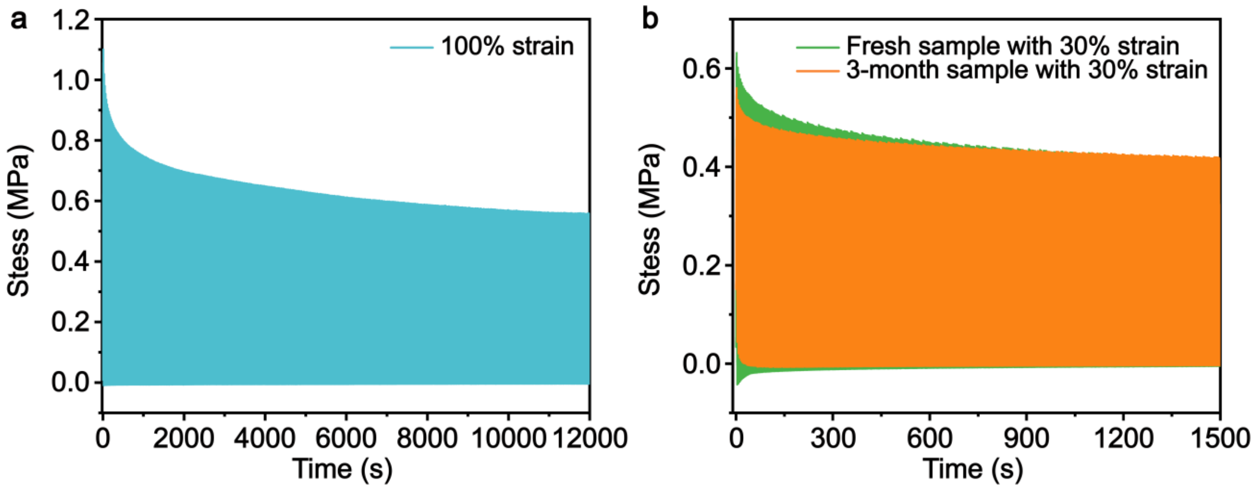

Supplementary Fig. 17. The reliability and long-term performance of DSICE materials. (**a**) Cyclic durability of DSICE-20 at 100% strain for 600 cycles (12000 s). (**b**) Cyclic stability of the fresh DSICE-20 and one stored for 3-month at 30% strain for 375 cycles (1500 s).

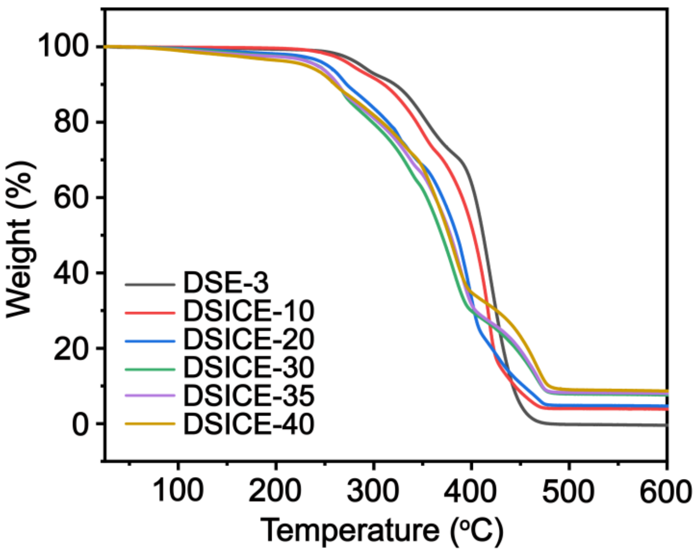

Supplementary Fig. 18. TGA traces of DSICE samples at 10 °C min^{-1} under N_2 atmosphere. It demonstrates excellent thermal stability of DSICE, of which possess high decomposition temperature, up to 230 °C.

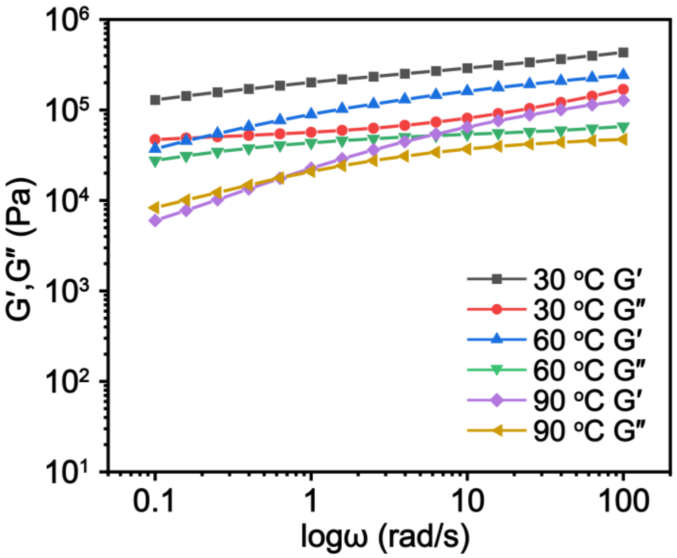

Supplementary Fig. 19. Storage modulus G′ and loss modulus G″ as functions of angular frequency ω for the DSICE-30 at different sweeping temperatures.

The operating temperature of DSICE-30 was assessed by rheological measurements upon a frequency sweep (Supplementary Fig. 19). When the temperature is low, such as 30 °C, G′ is much higher than G″, indicative of the perfectly elastic response of the materials. The crossover point of G′ and G″ appears at the sweep temperature over 60 °C, suggesting that the viscous response starts to happen. Furthermore, the crossover point moves to the high-frequency direction as the sweeping temperature increases to 90 °C, meaning that the obvious viscous behavior occurs in the DSICE system. In order to make DSICE behavior more elastically, it is suggested that the operating temperature do not exceed 90 °C.

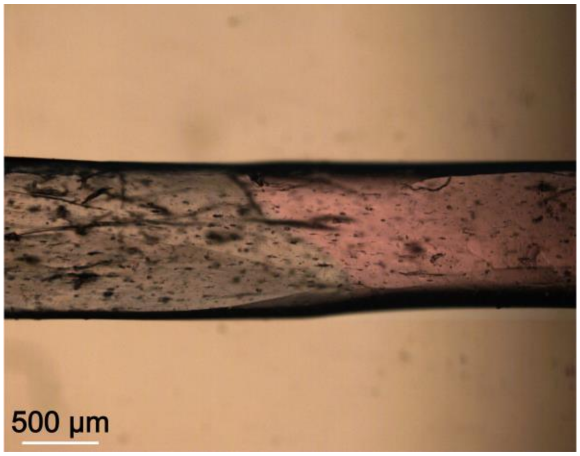

Supplementary Fig. 20. Optical microscopy image of the completely self-healed cut-off DSICE-30 samples. It indicates the seamless combination of two cut-off pieces after complete self-healing.

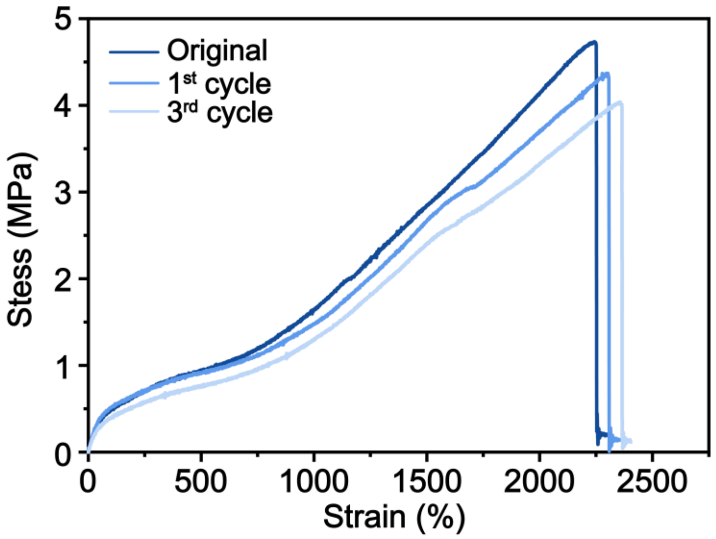

Supplementary Fig. 21. Typical stress-stain curves of DSICE-35 sample after three recycling. The stretching stress displayed a slight drop, which is attributed to the insufficient crosslinking of the UPy units after the recycling process.

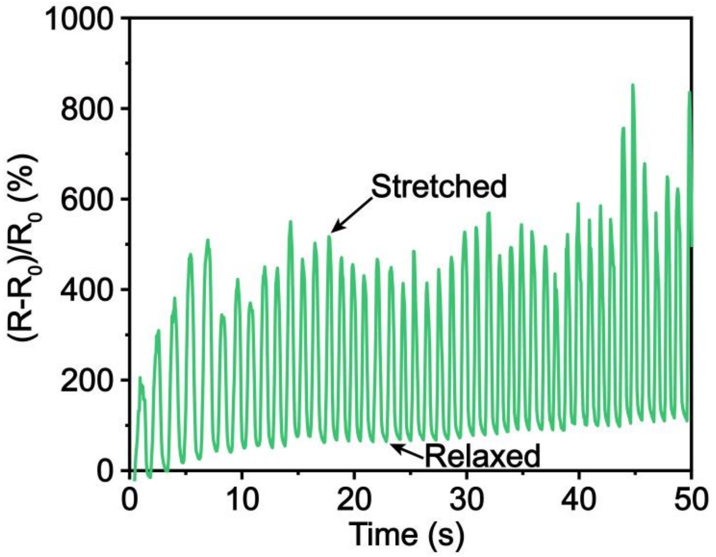

Supplementary Fig. 22. The impedance change of DSICE-30 sample when stretched to different tensile elongation and then relaxed time and again. It demonstrates the sensitive response of DSICE materials to different stretching strain.

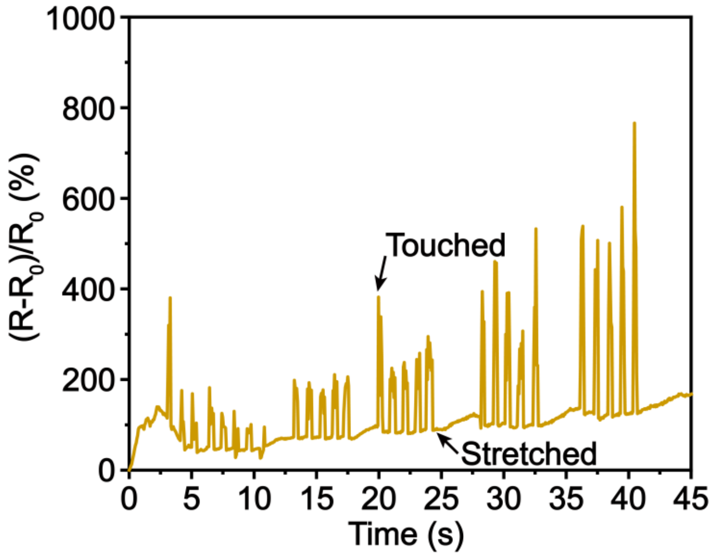

Supplementary Fig. 23. The impedance change of DSICE-30 sample at different touched stimuli when stretched to a fixed elongation. It indicates the sensitive response of DSICE materials to multiple stimuli.

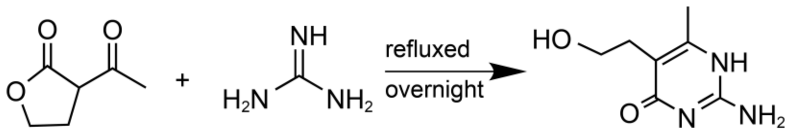

Supplementary Fig. 24. Synthesis of 2-ureido-4-pyrimidone (UPy). 9.9 g Guanidine carbonate (Aladdin, 99%) and 6 mL 2-Acetyl butyrolactone (Aladdin, 98%) were mixed and stirred in 60 mL anhydrous ethanol (RHAWN, AR, 99.7%). The mixture was refluxed overnight at 40 °C in the presence of 15.6 mL triethylamine (Aladdin, 99.0%). The suspension became yellow and turbid. After cooling, aqueous HCl (Acros, 37%) was used to adjust pH to 6-7. The white solid powder was filtered, washed with ethanol for three times and dried.

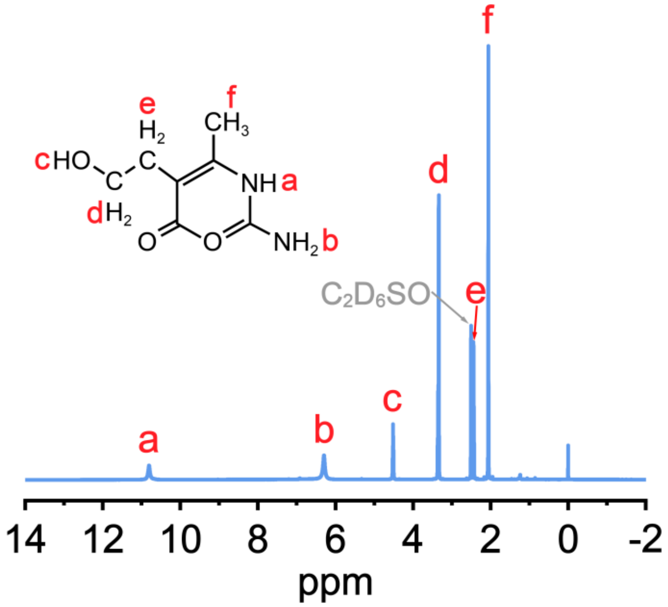

Supplementary Fig. 25. ^{1}H-NMR of 2-ureido-4-pyrimidone (UPy) in DMSO-d_6 (400 MHz). The hydrogen protons of UPy were observed at 10.8, 6.3, 4.5, 3.3, 2.4 and 2.1 ppm, which are respectively corresponding to the secondary amine, primary amine, hydroxyl, methylene and methyl groups.

Supplementary Tab. 1. Molecular weight of the synthesized DSE samples

Sample Code	Mn (g mol^{-1})	Mw (g mol^{-1})	Polymer Dispersity Index (PDI)
DSE-0	3.42×10^4	6.71×10^4	1.96
DSE-1	2.78×10^4	5.37×10^4	1.93
DSE-2	2.44×10^4	5.02×10^4	2.04
DSE-3	1.62×10^4	3.60×10^4	2.22

Supplementary Tab. 2. A rough comparison of the overall performance between this work and recently reported typical ionic conductor materials

Ionic Conductive Materials	Ionic conductivity (S m^{-1})	Mechanical Properties			Self-healing (r. t.)	Recyclability	Transparency (%)	Ref.
		Stretchability (%)	Strength (MPa)	Toughness (MJ m^{-3})				
DSICE	3.77×10^{-3}	2615.17	27.83	164.36	6 h, ~99%	Yes	92.3	This work
PAA/x-Ch ionogel	5.15×10^{-3}	1200	0.235	NA	None	None	Opaque	5
PBA ICE	10^{-4}~10^{-3}	1100	0.25	NA	None	None	92.1	8
P(SPMA-r-MMA) ionogel	4.1×10^{-2}	677	0.3	0.114	3 h 70.9%	None	>90	9
LM-PDES elastomer	1.3×10^{-2}	2600	0.125	2.36	24 h, ~98%	None	94	26
P(AAm/ChCl-co-MA/ChCl) elastomer	4.1×10^{-2}	450	0.6	NA	24 h 88.9%	None	95.1	27
PLA ICE	2.4×10^{-3}	800	0.18	1.3	15 h 90.1%	Yes	91.1	28
P(MEA-co-IBA) elastomer	4.20×10^{-4}	1640	1.05	0.039	24 h, ~30%	None	90	29
PIL elastomer	1.31×10^{-2}	540	0.24	NA	2 h ~100%	None	Opaque	46
PAA/betaine elastomer	2×10^{-5}	1600	~5	NA	12 h, ~100%	None	99.7	47
