# Supplementary material for: Phase-locked constructing dynamic supramolecular ionic conductive elastomers with superior toughness, autonomous self-healing and recyclability
Source: Nat Commun. 2022 Aug 18;13:4868. doi: 10.1038/s41467-022-32517-4 (PMC9388535; doi:10.1038/s41467-022-32517-4)
Supplement: Supplementary file 3 — Description of Additional Supplementary Files [file 41467_2022_32517_MOESM3_ESM.pdf]

Description of Additional Supplementary Files

File name: Supplementary Movie 1

Description: The stretching process of the intact DSE-3 sample and the pre-damaged one with 200 mm crack.

File name: Supplementary Movie 2

Description: Demonstration of excellent fast self-healing capability of DSICE-30 at ambient temperature. The dumbbell-shaped DSICE-30 film colored blue and red with standard 12 mm × 2 mm rectangular and a thickness of 0.5 mm was cut into two pieces, respectively. And then put any two colored pieces into contact for 5 min and stretched to a large strain >800%.

File name: Supplementary Movie 3

Description: Demonstration of the impedance changes of DSICE-30 when stretched to different tensile elongation.

File name: Supplementary Movie 4

Description: Demonstration of the impedance changes of DSICE-30 at different touched stimuli when stretched.
